# Recurrent Left Abducens Nerve Palsy and Idiopathic Intracranial Hypertension During Pregnancy: A Diagnostic Challenge

**DOI:** 10.7759/cureus.115680

**Published:** 2026-09-02

**Authors:** Tarun V Ramlogan, Aamir S Ali, Raenelle Ramlogan

**Affiliations:** 1 Internal Medicine, Sangre Grande Hospital, Sangre Grande, TTO; 2 Internal Medicine, Sangre Grande General, Sangre Grande, TTO

**Keywords:** abducens nerve palsy, acetazolamide, diplopia, idiopathic intracranial hypertension, lumbar puncture, pregnancy

## Abstract

Idiopathic intracranial hypertension (IIH) is characterized by elevated intracranial pressure without an identifiable mass lesion, hydrocephalus, cerebral venous sinus thrombosis, or abnormal cerebrospinal fluid composition. Diagnosis during pregnancy can be challenging because headache and visual symptoms overlap with several neurologic and obstetric disorders. We report a previously healthy 20-year-old primigravida with a body mass index of approximately 33 kg/m² who presented at 17 weeks’ gestation with intermittent headache, photophobia, blurred vision, horizontal diplopia, nasal deviation of the left eye, and absent left-eye abduction. Visual acuity was 6/6 bilaterally, with mild bilateral optic disc swelling, greater on the left. The initial brain magnetic resonance imaging report described subtle paired diffusion-weighted signal abnormalities in the region of the medial longitudinal fasciculi, interpreted as possible mild cytotoxic edema related to a potentially reversible ischemic event. These findings were not anatomically concordant with the documented isolated left abduction deficit, and subsequent informal radiologic review raised the possibility that they were artifactual; no formal radiology addendum was available. Magnetic resonance angiography showed no significant intracranial arterial abnormality, and magnetic resonance venography excluded cerebral venous sinus thrombosis. Lumbar puncture in the lateral decubitus position demonstrated an opening pressure of 31 cmH₂O with normal cerebrospinal fluid constituents, followed by resolution of her symptoms and abducens palsy after drainage. Symptoms recurred at 20 weeks, when repeat opening pressure was 42 cmH₂O; and they again improved after drainage. Acetazolamide was commenced at 250 mg twice daily and increased to 500 mg twice daily at 33 weeks because of persistent optic disc swelling. The patient underwent cesarean delivery at 36 weeks; the specific clinical rationale for the mode of delivery was unavailable in the records reviewed. The neonate weighed 2.6 kg, had Apgar scores of seven and eight, and had no documented neonatal complications. Acetazolamide was continued postpartum. At telephone review approximately six months after delivery, the patient remained clinically well, reported adherence to her prescribed acetazolamide, and denied recurrent headache, blurred vision, diplopia, or ocular deviation. Final objective ophthalmologic resolution could not be confirmed because she did not attend her most recent scheduled ophthalmology follow-up. This case emphasizes the need to integrate clinical examination, cerebrospinal fluid findings, and neuroimaging when evaluating intracranial hypertension during pregnancy.

## Introduction

Idiopathic intracranial hypertension (IIH) is a disorder characterized by elevated intracranial pressure in the absence of an intracranial mass lesion, hydrocephalus, cerebral venous sinus thrombosis, or abnormal cerebrospinal fluid composition [1,2]. It occurs predominantly in women of reproductive age and is strongly associated with obesity. Common clinical manifestations include headache, transient visual obscurations, blurred vision, pulsatile tinnitus, and papilledema. Diplopia may also occur, most commonly due to abducens nerve dysfunction, which is recognized as a false-localizing sign of raised intracranial pressure [1,2].

The diagnosis of IIH requires careful exclusion of secondary causes of raised intracranial pressure. Neuroimaging, including venous imaging, is essential to exclude structural intracranial pathology and cerebral venous sinus thrombosis, while lumbar puncture typically demonstrates an elevated opening pressure with otherwise normal cerebrospinal fluid constituents [1,2]. Diagnosis can be particularly challenging during pregnancy because headache and visual symptoms may also occur with hypertensive disorders of pregnancy, including preeclampsia, cerebral venous sinus thrombosis, ischemic disease, demyelinating conditions, and other structural or neurologic disorders [3,4]. Distinguishing IIH from these secondary causes is important because management and maternal-fetal implications may differ considerably. Preservation of visual function is a major priority, making early ophthalmologic assessment and coordinated neurologic, obstetric, ophthalmologic, and radiologic evaluation important.

Management during pregnancy requires an individualized approach that balances maternal neurologic and ophthalmologic outcomes with potential fetal risks. Therapeutic lumbar puncture may provide temporary symptomatic relief, while acetazolamide is commonly used to reduce cerebrospinal fluid production when ongoing medical therapy is required. Its use during pregnancy should follow an individualized risk-benefit discussion involving the patient and obstetric team [3,5,6].

We report a pregnant patient with recurrent left abducens nerve palsy, bilateral optic disc swelling, and markedly elevated cerebrospinal fluid opening pressures on two separate lumbar punctures, with symptomatic improvement following cerebrospinal fluid drainage. The recurrence of an isolated abducens nerve palsy in association with objectively demonstrated intracranial hypertension during pregnancy represents an unusual clinical presentation. Diagnostic evaluation was further complicated by subtle paired midbrain diffusion-weighted signal abnormalities described in the initial magnetic resonance imaging report that were not anatomically concordant with the documented isolated left abduction deficit. Subsequent informal radiologic review raised the possibility that these findings were artifactual, although no formal radiology addendum was available. The case therefore illustrates both the importance of excluding pregnancy-associated secondary causes of raised intracranial pressure and the need to reconcile potentially discordant neuroimaging findings with the clinical neurologic examination.

## Case presentation

A previously healthy 20-year-old primigravida at 17 weeks’ gestation presented with a one-week history of intermittent headaches and abnormal deviation of the left eye. The headaches were associated with photophobia, blurred vision, and horizontal diplopia. Her pregnancy had otherwise been uncomplicated, and antenatal anomaly scans were reported as normal. She had no previous neurologic or other significant medical history.

Blood pressure was documented as normal at presentation, and urinalysis demonstrated no proteinuria, making a hypertensive disorder of pregnancy less likely. Hemoglobin was 13.8 g/dL, with no evidence of anemia. Baseline hematologic, renal, hepatic, electrolyte, metabolic, and autoimmune investigations were otherwise unremarkable. The white blood cell count was 9.39 ×10⁹/L, which was within the expected physiologic range for pregnancy. Baseline laboratory investigations are summarized in Table 1.

**Table 1:** Baseline laboratory investigations g/dL, grams per deciliter; ×10⁹/L, 10⁹ cells per liter; mmol/L, millimoles per liter; mg/dL, milligrams per deciliter; U/L, units per liter; IU/mL, international units per milliliter; C3, complement component 3; C4, complement component 4.

Investigation	Patient result	Reference range	Unit
Hemoglobin	13.8	12.9-15.9	g/dL
White blood cell count	9.39	3.7-10.10	×10⁹/L
Platelet count	281	155-366	×10⁹/L
Sodium	137	135-145	mmol/L
Potassium	3.9	3.6-5.2	mmol/L
Urea	5	2.1-7.1	mmol/L
Creatinine	0.5	0.5-1.20	mg/dL
Alkaline phosphatase	56	35-104	U/L
Aspartate aminotransferase	11	8-43	U/L
Urinalysis	Normal; no proteinuria	Normal / negative	—
Complement C3	125	75-175	mg/dL
Complement C4	39	14-40	mg/dL
Rheumatoid factor	7.95	<14.0	IU/mL

Ocular examination demonstrated nasal deviation of the left eye with absent left-eye abduction and horizontal diplopia, consistent with a left abducens nerve palsy. Visual acuity was 6/6 bilaterally, and color vision was preserved. Ophthalmologic assessment documented mildly raised appearances of both optic discs, more pronounced on the left. The remainder of the neurologic examination was unremarkable.

Neuroimaging

Magnetic resonance imaging (MRI) of the brain, magnetic resonance angiography (MRA), and magnetic resonance venography (MRV) were performed. The initial MRI report described subtle paired mildly hyperintense foci on diffusion-weighted imaging in the region of the medial longitudinal fasciculi within the ventral periaqueductal gray matter of the midbrain. These were interpreted as possible mild cytotoxic edema related to a potentially reversible ischemic event. No demyelinating lesion, previous hemorrhagic change, focal mass effect, hydrocephalus, or other significant structural intracranial abnormality was identified. The orbits, optic nerves, and extraocular muscles were reported as unremarkable. MRA did not identify a significant intracranial arterial abnormality, while MRV demonstrated patent cerebral venous sinuses without evidence of cerebral venous sinus thrombosis.

The reported midbrain finding was not anatomically concordant with the documented isolated absence of left-eye abduction. Following subsequent informal review and discussion of the original images with a radiologist, the previously reported paired medial longitudinal fasciculus abnormalities were not considered convincing, and the subtle diffusion-weighted appearance was considered potentially artifactual in the context of an otherwise essentially normal MRI and the clinical findings. However, no formal radiology addendum or documented targeted neuroradiology report was issued. Because no formal addendum was available, the initial report remained the documented radiologic interpretation, and the clinical significance of the reported diffusion abnormality remained uncertain. No follow-up MRI was available to demonstrate persistence or resolution of the reported finding.

The diffusion-weighted image, corresponding apparent diffusion coefficient map, and magnetic resonance venographic image are shown in Figures 1-3.

**Figure 1:**
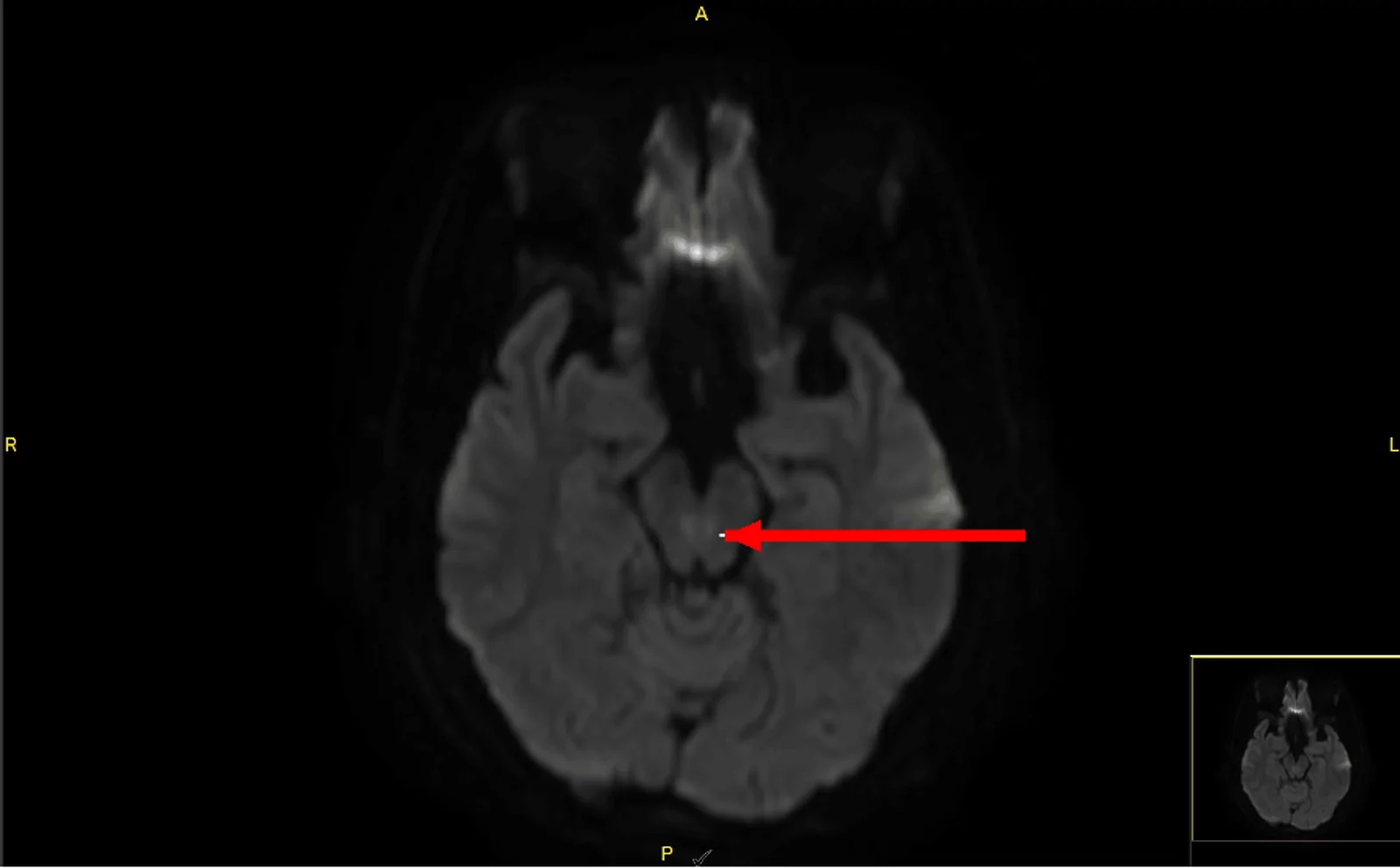
Axial diffusion-weighted magnetic resonance image (b=1000) The arrow indicates the periaqueductal midbrain region in which subtle paired signal abnormalities involving the medial longitudinal fasciculi were described in the initial radiology report. The clinical significance of this finding remains uncertain.

**Figure 2:**
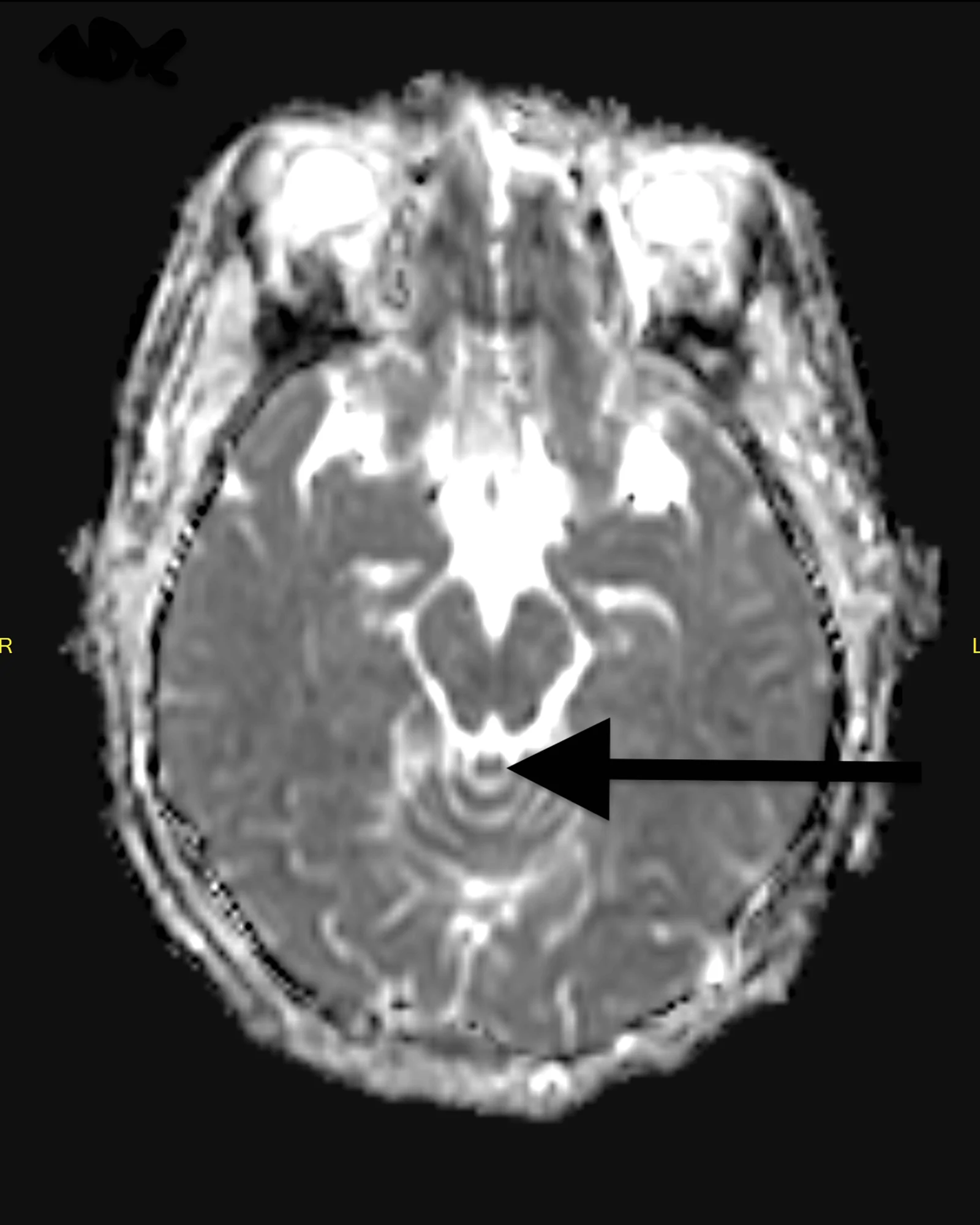
Corresponding axial apparent diffusion coefficient map The arrow indicates the region corresponding to the diffusion-weighted imaging finding described in the initial radiology report. Definitive confirmation of true restricted diffusion was not available.

**Figure 3:**
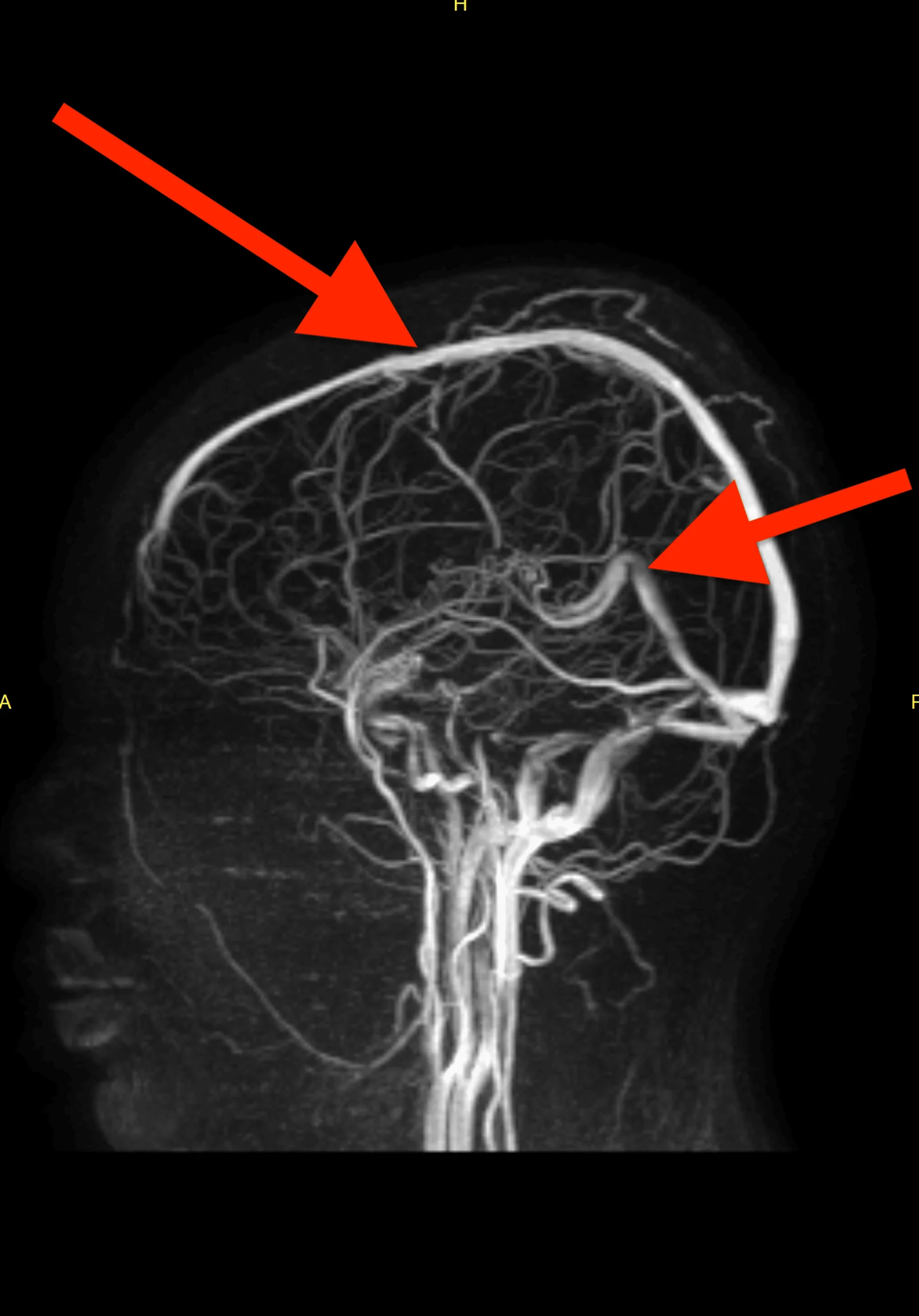
Sagittal magnetic resonance venography The arrows indicate patent major cerebral venous sinuses, including the superior sagittal and posterior transverse-sigmoid venous pathways; no cerebral venous sinus thrombosis was identified.

First lumbar puncture

At 17 weeks’ gestation, a diagnostic and therapeutic lumbar puncture was performed with the patient in the lateral decubitus position. The opening pressure was 31 cmH₂O and decreased to a closing pressure of 21 cmH₂O following cerebrospinal fluid drainage. The cerebrospinal fluid was clear and colorless, without xanthochromia. There were no polymorphs, lymphocytes, or red blood cells. Cerebrospinal fluid glucose was 62 mg/dL, lactate was 15 mg/dL, and total protein was 16 mg/dL. No organisms were identified on Gram staining.

Following cerebrospinal fluid drainage, the headache, blurred vision, and horizontal diplopia resolved. By the following day, the left abducens nerve palsy had resolved, and extraocular movements were documented as normal.

Recurrence and second lumbar puncture

Approximately two weeks later, at 20 weeks’ gestation, the patient presented once again with recurrent nasal deviation of the left eye, horizontal diplopia, headache, and blurred vision. Repeat examination was consistent with recurrence of the left abducens nerve palsy.

A second diagnostic and therapeutic lumbar puncture was performed in the lateral decubitus position. The opening pressure was 42 cmH₂O and decreased to a closing pressure of 22 cmH₂O following cerebrospinal fluid drainage. Cerebrospinal fluid analysis again demonstrated no polymorphs, lymphocytes, or red blood cells. Glucose was 60 mg/dL, lactate was 15 mg/dL, and total protein was 13 mg/dL. Gram staining showed no organisms. Her symptoms and ocular deviation again improved following cerebrospinal fluid drainage.

The combination of bilateral optic disc swelling, markedly elevated opening pressures on two separate lumbar punctures, cerebrospinal fluid findings without evidence of an inflammatory or infectious process, absence of a mass lesion or hydrocephalus, and exclusion of cerebral venous sinus thrombosis provided strong evidence of intracranial hypertension. Following consideration of relevant secondary causes, the treating team made a clinical diagnosis of IIH. The significance of the initially reported midbrain diffusion abnormality remained a diagnostic consideration because of the clinical-radiologic discrepancy described above. The lumbar puncture pressures and cerebrospinal fluid laboratory findings are summarized in Table 2.

**Table 2:** Lumbar puncture pressures and cerebrospinal fluid findings Reference values shown are commonly used adult cerebrospinal fluid ranges and may vary by laboratory. Units: cmH₂O, centimeters of water; cells/µL, cells per microliter; mg/dL, milligrams per deciliter

Parameter	First lumbar puncture (17 weeks)	Second lumbar puncture (20 weeks)	Reference value
Opening pressure	31 cmH₂O	42 cmH₂O	≤25 cmH₂O in adults
Closing pressure	21 cmH₂O	22 cmH₂O	No fixed diagnostic reference; interpreted clinically
Polymorphs	Nil	Nil	0-5 cells/µL total white blood cells
Lymphocytes	Nil	Nil	0-5 cells/µL total white blood cells
Red blood cells	Nil	Nil	0 cells/µL
Glucose	62 mg/dL	60 mg/dL	40-80 mg/dL
Lactate	15 mg/dL	15 mg/dL	Approximately 10-22 mg/dL
Total protein	16 mg/dL	13 mg/dL	15-45 mg/dL
Gram stain	No organisms	No organisms	No organisms

Treatment and antenatal follow-up

Following multidisciplinary discussion with the obstetric team, acetazolamide was commenced at 20 weeks’ gestation at a dose of 250 mg orally twice daily. At subsequent neurology outpatient review, the patient remained clinically well, reported adherence to treatment, and had no new neurologic symptoms. Repeat laboratory investigations, including renal function and serum electrolytes, remained within normal limits, and no medication-related adverse effects were documented in the available records.

At 33 weeks’ gestation, ophthalmologic review documented persistent optic disc swelling, particularly involving the left optic disc. Optical coherence tomography (OCT) was reported to demonstrate bilateral optic nerve-head swelling. The original OCT images, quantitative measurements, formal visual-field results, and full serial ophthalmology notes were unavailable. In view of persistent optic disc swelling, acetazolamide was increased to 500 mg orally twice daily.

Delivery and postpartum outcome

At 36 weeks’ gestation, the patient underwent cesarean delivery. Although a recommendation for cesarean delivery from the treating neurologist was documented, the specific clinical rationale for the mode of delivery was not available in the records reviewed. Therefore, the mode of delivery could not be attributed to IIH itself.

The neonate had Apgar scores of seven at one minute and eight at five minutes and a birth weight of 2.6 kg. No neonatal complications were documented.

Acetazolamide was continued postpartum, with ongoing neurology and ophthalmology follow-up. At telephone review approximately six months after delivery, the patient remained clinically well and reported adherence to acetazolamide 500 mg orally twice daily. She denied recurrent headache, blurred vision, horizontal diplopia, ocular deviation, or other new neurologic symptoms.

The six-month telephone review was reassuring clinically; however, it did not provide an objective ophthalmologic examination. The patient subsequently did not attend her most recent scheduled ophthalmology follow-up. Therefore, updated visual acuity, optic disc appearance, visual-field assessment, and objective confirmation of resolution or persistence of the previously reported OCT optic nerve-head swelling were unavailable. A chronological summary of the clinical course is provided in Table 3.

**Table 3:** Clinical timeline

Gestational age/time	Clinical event
17 weeks	Presented with headache, photophobia, blurred vision, horizontal diplopia, nasal deviation of the left eye, and absent left-eye abduction.
17 weeks	Mild bilateral optic disc swelling documented. Magnetic resonance imaging, magnetic resonance angiography, and magnetic resonance venography were performed; cerebral venous sinus thrombosis was excluded.
17 weeks	First lumbar puncture: opening pressure 31 cmH₂O and closing pressure 21 cmH₂O; cerebrospinal fluid findings were otherwise unremarkable; symptoms and abducens palsy resolved after drainage.
20 weeks	Re-presented with recurrent left abducens nerve palsy and visual symptoms.
20 weeks	Second lumbar puncture: opening pressure 42 cmH₂O and closing pressure 22 cmH₂O; cerebrospinal fluid findings were otherwise unremarkable; symptoms improved after drainage.
20 weeks	Acetazolamide 250 mg orally twice daily commenced following obstetric discussion.
33 weeks	Persistent optic disc swelling and optical coherence tomography-reported bilateral optic nerve-head swelling; acetazolamide increased to 500 mg orally twice daily.
36 weeks	Cesarean delivery; although a neurologic recommendation was documented, the precise clinical rationale for the mode of delivery was unavailable. Neonatal birth weight 2.6 kg; Apgar scores 7 and 8; no documented neonatal complications.
Postpartum	Acetazolamide 500 mg orally twice daily continued, with planned neurology and ophthalmology follow-up.
Approximately 6 months postpartum	Telephone review: clinically well, asymptomatic, and adherent to acetazolamide; no recurrent headache, diplopia, blurred vision, or ocular deviation.
Most recent scheduled ophthalmology follow-up	Patient did not attend; final objective ophthalmologic outcome remained unavailable.

## Discussion

This case highlights several important clinical considerations. Although headache and visual disturbance are common during pregnancy, the combination of horizontal diplopia, an ocular motility deficit, and optic disc swelling should prompt urgent evaluation for raised intracranial pressure and secondary neurologic causes. In this patient, nasal deviation of the left eye, absent abduction, and horizontal diplopia were clinically consistent with a left abducens nerve palsy. Abducens nerve palsy is a recognized false-localizing sign of raised intracranial pressure because the nerve’s long intracranial course makes it susceptible to stretching or displacement [1,2].

The evidence for intracranial hypertension was substantial. Bilateral optic disc swelling was documented, and lumbar punctures performed in the lateral decubitus position demonstrated opening pressures of 31 cmH₂O and 42 cmH₂O, both exceeding the adult diagnostic threshold of 25 cmH₂O [1,2]. Cerebrospinal fluid findings were otherwise unremarkable, with no pleocytosis or evidence of an infectious or inflammatory process, and magnetic resonance venography excluded cerebral venous sinus thrombosis. The patient’s headache, visual symptoms, and ocular motor deficit improved following cerebrospinal fluid drainage, with recurrence associated with a higher opening pressure and subsequent improvement after repeat drainage. Although response to lumbar puncture is not independently diagnostic, this reproducible clinical course further supported raised intracranial pressure as the principal mechanism underlying her presentation.

The diagnosis of IIH requires exclusion of secondary causes of intracranial hypertension, which is particularly important during pregnancy [1,2]. In this patient, blood pressure was documented as normal and urinalysis showed no proteinuria, making a hypertensive disorder of pregnancy less likely. Hemoglobin was 13.8 g/dL, with no evidence of anemia, while renal, hepatic, electrolyte, and metabolic investigations were unremarkable. Neuroimaging demonstrated no intracranial mass lesion or hydrocephalus, and magnetic resonance venography excluded cerebral venous sinus thrombosis. These findings, together with otherwise unremarkable cerebrospinal fluid findings and markedly elevated opening pressures, supported the treating team’s diagnosis of IIH. However, the interpretation of the initially reported midbrain diffusion abnormality required careful consideration before the process could confidently be regarded as idiopathic.

The initial MRI interpretation represented the principal clinical-radiologic discrepancy in this case. The original report described subtle paired diffusion-weighted abnormalities in the region of the medial longitudinal fasciculi within the ventral periaqueductal midbrain and interpreted these as possible mild cytotoxic edema related to a potentially reversible ischemic event. However, lesions of the medial longitudinal fasciculus classically produce internuclear ophthalmoplegia, characterized predominantly by impaired adduction of the ipsilateral eye with abducting nystagmus of the contralateral eye [7]. This differs anatomically from the isolated absence of left-eye abduction documented in this patient, which was clinically consistent with an abducens nerve palsy.

Following subsequent informal review and discussion of the original MRI images with a radiologist, the previously reported paired medial longitudinal fasciculus abnormalities were not considered convincing, and the MRI brain was regarded as otherwise essentially normal. In the context of the clinical findings and the lack of an anatomically compatible ocular motor deficit, the subtle diffusion-weighted appearance was considered potentially artifactual. However, no formal radiology addendum or documented targeted neuroradiology report was issued, and no follow-up MRI was available to demonstrate persistence or resolution of the reported signal abnormality. Because no formal radiology addendum was issued, the initial report remained the documented radiologic interpretation, and the finding could not be definitively reclassified as artifact. This distinction is important because strict diagnostic frameworks for IIH require the absence of a structural or vascular lesion capable of adequately explaining the clinical syndrome [1]. The clinical-radiologic discrepancy and the importance of reconciling imaging findings with the neurologic examination therefore represent central teaching points of this case.

Pregnancy-specific management of IIH is guided primarily by the severity of optic disc swelling, visual dysfunction, headache burden, and risk of progressive visual loss. Therapeutic lumbar puncture can provide temporary symptomatic relief and may be used as a bridging measure during pregnancy, although repeated procedures are not considered a durable long-term treatment strategy [2-4]. Contemporary reviews emphasize multidisciplinary care and individualized management based on visual risk and maternal-fetal considerations [8,9]. In this patient, both lumbar punctures produced prompt symptomatic improvement, but recurrence after the first procedure illustrated the temporary nature of cerebrospinal fluid drainage.

Acetazolamide decreases cerebrospinal fluid production and is widely used in IIH. In nonpregnant adults with mild visual loss, the Idiopathic Intracranial Hypertension Treatment Trial demonstrated modest improvement in visual-field function and papilledema when acetazolamide was combined with a weight-reduction diet [5]. Its use during pregnancy remains individualized. Pregnancy-focused guidance recommends discussion with the patient and obstetric team because historical concerns regarding teratogenicity have largely arisen from animal data, while available human observational evidence has not demonstrated a convincing increase in adverse pregnancy outcomes [3,6,8,9]. In this case, acetazolamide was commenced after the first trimester following obstetric consultation and was subsequently increased because of persistent optic disc swelling.

The patient underwent cesarean delivery at 36 weeks. Although a neurologic recommendation for cesarean delivery was documented, the precise clinical rationale for the mode of delivery was unavailable in the records reviewed and should not be inferred retrospectively. Importantly, IIH itself is not generally considered an indication for cesarean delivery. In patients with stable disease or disease in remission, vaginal delivery is usually possible, with the mode of delivery determined primarily by obstetric considerations unless specific neurologic or ophthalmologic circumstances dictate otherwise [3,4,8,9]. The absence of a documented indication for cesarean delivery therefore remains a limitation of this report.

Clinical improvement should not replace objective ophthalmologic surveillance. Persistent bilateral optic nerve-head swelling was reported on optical coherence tomography at 33 weeks despite symptomatic improvement, prompting escalation of acetazolamide. Optical coherence tomography provides objective structural assessment of the optic nerve head and retinal nerve fiber layer and has an important role in longitudinal assessment of IIH-related optic nerve changes [10]. At telephone review approximately six months postpartum, the patient remained clinically well and asymptomatic while continuing acetazolamide. However, because this assessment was conducted by telephone and she did not attend her most recent scheduled ophthalmology follow-up, objective confirmation of resolution of the optic disc swelling was unavailable.

This case has several limitations. Complete serial ophthalmology documentation, quantitative optical coherence tomography measurements, formal visual-field results, and the original optical coherence tomography images were unavailable. The final postpartum assessment was clinical rather than ophthalmologic, and the patient did not attend her most recent scheduled eye review. The precise indication for cesarean delivery was also not documented. In addition, although subsequent informal radiologic review raised the possibility that the initially reported midbrain diffusion abnormality represented artifact, no formal radiology addendum or documented targeted neuroradiology report was available, and no follow-up MRI was performed. Consequently, the imaging finding cannot be definitively reclassified. Despite these limitations, the combination of bilateral optic disc swelling, two markedly elevated opening pressures, cerebrospinal fluid findings without evidence of an inflammatory or infectious process, exclusion of cerebral venous sinus thrombosis, absence of a mass lesion or hydrocephalus, and reproducible clinical improvement following cerebrospinal fluid drainage provides strong evidence for intracranial hypertension and was considered supportive of the treating team’s diagnosis of IIH, while the unresolved initial imaging finding warrants continued caution regarding a strictly idiopathic classification. Recent reviews continue to emphasize multimodal assessment, preservation of visual function, and longitudinal ophthalmologic surveillance in IIH [11].

## Conclusions

Raised intracranial pressure should be considered in pregnant patients presenting with headache, horizontal diplopia, and an abducens nerve palsy. In this patient, recurrent ocular symptoms were associated with markedly elevated opening pressures and improved following therapeutic cerebrospinal fluid drainage. Acetazolamide was introduced after multidisciplinary discussion and later increased because of persistent optic disc swelling.

This case also demonstrates the importance of reconciling neuroimaging findings with the neurologic examination. The medial longitudinal fasciculus abnormality described in the initial MRI report was not anatomically concordant with the documented isolated left abduction deficit, and subsequent informal radiologic review raised the possibility that the finding was artifactual. In the absence of a formal radiology addendum, the initial report remained the documented radiologic interpretation and the imaging discrepancy could not be definitively resolved. Although the overall clinical findings strongly supported intracranial hypertension and the treating team’s diagnosis of IIH, the unresolved initial imaging finding warrants caution regarding a strictly idiopathic classification. Continued objective ophthalmologic surveillance remains important even when symptoms improve, as illustrated by the patient remaining asymptomatic at approximately six months postpartum despite the absence of a definitive later ophthalmologic assessment.
